# Etanercept Prevents Endothelial Dysfunction in Cafeteria Diet-Fed Rats

**DOI:** 10.3390/ijerph19042138

**Published:** 2022-02-14

**Authors:** Scăunaşu Răzvan-Valentin, Sertaç Ata Güler, Tijen Utkan, Tuğçe Demirtaş Şahin, Gulcin Gacar, Yusufhan Yazir, Selenay Furat Rencber, Lupușoru Mircea, Bălălău Cristian, Popescu Bogdan, Nihat Zafer Utkan

**Affiliations:** 1 Department of General Surgery, Faculty of General Medicine, “Coltea” Hospital, Carol Davila University, 020021 Bucharest, Romania; mircealupusoru@yahoo.com (L.M.); dr.balalau@gmail.com (B.C.); dr.bpopescu@gmail.com (P.B.); 2 Department of General Surgery, Medical Faculty, Kocaeli University, Kocaeli 41380, Turkey; zaferutkan@yahoo.com; 3 Department of Pharmacology, Medical Faculty, Kocaeli University, Kocaeli 41380, Turkey; tijenutkan@hotmail.com (T.U.); tugcemedusa@gmail.com (T.D.Ş.); 4 Experimental Medical Research and Application Center, Kocaeli University, Kocaeli 41380, Turkey; 5 Stem Cell and Gene Therapy Research and Application Center, Kocaeli University, Kocaeli 41380, Turkey; gulcingacar@gmail.com (G.G.); yusufhanyazir@yahoo.com (Y.Y.); 6 Department of Histology and Embryology, Medical Faculty, Kocaeli University, Kocaeli 41380, Turkey; selenay.furat@hotmail.com

**Keywords:** cafeteria diet, endothelial dysfunction, etanercept, inflammation, nitric oxide TNF-α

## Abstract

Obesity is associated with endothelial dysfunction and this relationship is probably mediated in part by inflammation. **Objective:** The current study evaluated the effects of etanercept, a tumor necrosis factor-alpha (TNF-α) inhibitor, on endothelial and vascular reactivity, endothelial nitric oxide synthase (eNOS) immunoreactivity, and serum and aortic concentrations of TNF-α in a diet-induced rat model. **Design and results:** Male weanling Wistar rats were exposed to a standard diet and cafeteria diet (CD) for 12 weeks and etanercept was administered during CD treatment. Isolated aortas of the rats were used for isometric tension recording. Carbachol-induced relaxant responses were impaired in CD-fed rats, while etanercept treatment improved these endothelium-dependent relaxations. No significant change was observed in papaverine- and sodium nitroprusside (SNP)-induced relaxant responses. eNOS expression decreased in CD-fed rats, but no change was observed between etanercept-treated CD-fed rats and control rats. CD significantly increased both the serum and the aortic levels of TNF-α, while etanercept treatment suppressed these elevated levels. CD resulted in a significant increase in the body weight of the rats. Etanercept-treated (ETA) CD-fed rats gained less weight than both CD-fed and control rats.

## 1. Introduction

Obesity is a worldwide health problem associated with cardiovascular diseases, type 2 diabetes, chronic inflammation, and cancer [1]. It is also characterized by detrimental effects on vasculature including reduced endothelial function in different vascular beds [2,3,4]. Endothelial dysfunction, an independent predictor for the initiation and progression of atherosclerosis, has been established as a key mediator that links obesity with cardiovascular diseases [5].

The vascular endothelium plays a crucial role in modulating vascular tone and vasomotor function and is also involved in the regulation of inflammation, platelet aggregation, and thrombosis. Nitric oxide (NO) is the major mediator of these functions and physiological processes [6]. Endothelial dysfunction is generally associated with reduced NO bioavailability [7]. Recently, it has been demonstrated that vascular inflammation secondary to elevated levels of proinflammatory cytokines, such as tumor necrosis factor alpha (TNF-α), plays a crucial role in endothelial dysfunction [8]. Furthermore, TNF-α-mediated inflammation, which contributes to the development of many cardiovascular dysfunctions such as chronic heart failure, sepsis-associated cardiovascular disorders, myocardial ischemia-reperfusion injury, and atherosclerosis [9,10,11] reduces the release of NO and impairs NO-mediated vasorelaxation in the endothelium [12] by the induction of inducible nitric oxide synthase (iNOS) activation and reactive oxygen species (ROS) generation or by reducing the expression of endothelial nitric oxide synthase (eNOS), which is the primary enzyme involved in NO generation in vascular tissues [8,13]. Moreover, previous studies demonstrated that TNF-α decreased eNOS expression by inhibiting gene promoter activity [14].

The cafeteria diet (CD) is a diet-induced obesity model, generally used in animal studies because of its similarities with human obesity [15]. Several lines of evidence revealed that obesity is accompanied by chronic inflammation due to the overproduction of proinflammatory cytokines, including TNF-α, which impairs vascular reactivity [16]. It is documented that aortic tissues from obese Zucker rats showed an increased expression of TNF-α in vascular tissue, indicating that obesity is associated with vascular inflammation [17]. Moreover, overproduction of vascular TNF-α impaired vascular reactivity by reducing NO bioavailability in small arteries from visceral fat of obese patients [18]. These findings suggest that pharmacological strategies that target inflammation pathways may be therapeutically useful in treating obesity-related endothelial dysfunction. We previously reported that etanercept (an anti-TNF-α agent that binds to and functionally inactivates TNF-α) improved endothelial function in rats with diabetes mellitus [19], however, its effects on obesity-related vascular dysfunction remain unclear.

Based on this knowledge, the goal of the current study was to evaluate the effects of chronic etanercept treatment on endothelial and vascular reactivity, eNOS immunoreactivity, and serum and aortic concentrations of TNF-α in CD-fed rats.

## 2. Materials and Methods

### 2.1. Animal Preparation and Experimental Design

The study experiments utilized weanling male (30 day-old) Wistar albino rats (50–70 g) kept in a humidity- and temperature-controlled room (62 ± 7% and 22 ± 3 °C) with a 12-h light/dark cycle. The rats were given tap water and food pellets without any restrictions. It is important to note that the current study is in line with the Rules of Animal Research Ethics Committee (Number 26220, 6 July 2006) for animal care, and that it also received approval from the Animal Research Ethics Committee.

Rats were separated into the three groups (each *n* = 10): control (not exposed to CD), CD (exposed to CD for 12 weeks), and CD + ETA (exposed to CD and treated with subcutaneous injections of etanercept (0.8 mg/kg/week) for 12 weeks). The control group was fed with a standard pellet diet. The CD and CD + ETA groups were fed with CD, which is a high-fat diet containing a mixture of pate, chocolate, cookies, chow, bacon, potato chips and chow with proportions of 2:1:1:1:1:1, respectively, as previously published and these were given to each rat daily [20]. The composition of this cafeteria diet was 10% of energy as protein, 35.4% as carbohydrates, and 54.6% as lipids by dry weight (399 kcal/100 g in the cafeteria diet vs. 265 kcal/100 g in the pelleted diet). There were no restrictions on food amount eaten. Food intake and body weight were recorded weekly. After a 12-week treatment period, animals were sacrificed, the aortas of the rats were removed, and cumulative concentration-response curves of carbachol, sodium nitroprusside (SNP), and papaverine were obtained. Aortic eNOS and TNF-α expression were analyzed. Also, blood was collected and circulating levels of TNF-α were measured.

### 2.2. Organ Bath Studies

The thoracic aortas were quickly insulated after 12 weeks had passed. Initially placed in Krebs’ solution, washed, and cut into rings, the tissues were subsequently mounted in oxygenated (5% CO_2_/95% O_2_) organ baths containing 20 mL of Krebs’ solution (37 °C, pH 7.4) that consisted of (mM): NaCl, 118; KCl, 4.71; MgCl_2_, 1.05; NaH_2_PO_4_, 1.33; NaHCO_3_, 25; CaCl_2_, 2.7 and glucose, 5.6. A timeframe of 60 min was set to allow the tissues to properly equilibrate before starting the experiments. Each ring was set at 1 g in terms of prime resting tension and connected to a force-displacement transducer with the purpose of measuring the isometric tension. Recording the isometric tension entailed the utilization of a data acquisition system (MP30B-CE, Biopac, Biopac Systems, Inc. Santa Barbara, CA, USA), and software storage (BSL Pro 3.7, Biopac Systems) for data examination.

### 2.3. Agonist-Induced Contractions

Following the equilibration period, the feasibility of the preparation was tested by exposing the rings to KCl (80 mM). As part of the next step, the tissues were rinsed twice with Krebs’ solution and kept like that for 30 min to regain tension to the precontracted level. Once the 30 min had passed, each ring was progressively tightened with phenylephrine concentrations (10^−9^–10^−4^ M).

### 2.4. Agonist-Induced Relaxations

Arterial rings were primarily tightened using phenylephrine (3 × 10^−6^–10^−5^ M). The concentrations of phenylephrine were selected specifically to attain 85–87% of the maximum contraction. Once this had reached a plateau, carbachol (10^−8^–10^−5^ M), SNP (10^−8^–10^−4^ M), and papaverine (10^−5^–10^−4^ M) were added progressively to achieve the relaxation reactions. The mixture was substituted with a fresh one and the tissues were left for a 30 min timeframe to return the tension to the basal intensities between consecutive concentration-responsive curves.

### 2.5. Solutions and Drugs

Phenylephrine hydrochloride, carbachol chloride, SNP, and papaverine hydrochloride (Sigma Chemical, St. Louis, MO, USA) were dissolved in distilled water and prepared immediately before the start of the experiments. Etanercept (Wyeth, Münster, Germany) was administered to the rats at a volume of 0.1 mL per 100 g body weight, dissolved in physiological saline, and prepared rapidly prior to use.

### 2.6. Immunohistochemical Analyses

Following the 12-week treatment, the laboratory rats were decapitated under ketamine/xylazine (90/10 mg/kg) anesthesia while aortic tissues were insulated. To start, the tissues were secured in neutral-buffered formalin (10%) and implanted in paraffin wax. They were later cut into 3 µm units on a microtome, deparaffinized in xylene, and soaked in ethanol. The antigen was retrieved using a microwave oven (3–5 min, 600 W) and citrate protection. Endogenous peroxidase was blocked in methanol with H_2_O_2_ (3%) for a timeframe of 15 min and subsequently cleaned again. In the next step of the process, segments were incubated with a primary anti-eNOS rabbit polyclonal antibody (ab119292, Abcam, Cambridge, UK). These units were later incubated with a secondary biotinylated antibody, streptavidin–peroxidase, and a diaminobenzidine mixture. The segments were counterstained with Mayer’s hematoxylin and later fixed with entellan on glass slides. With the help of a light microscope, the samples were reproduced in images and photographed with a Leica DMC2900 device (CH-9435, Heerbrugg, Germany). The eNOS positive area% was analyzed with Image J software (U.S. National Institute of Health, Bethesda, MD, USA).

### 2.7. Enzyme-Linked Immunosorbent Assay (ELISA)

At the end of the 12-week etanercept administration, blood samples were collected to determine the circulating levels of TNF-α. The serum was segregated by centrifugation at 4000 rpm for 15 min at 40 °C and it was divided into aliquots and kept at −70 °C before the start of the experiments. Serum levels of the TNF-α were measured by ELISA kit (Biosource, Invitrogen, Carlsbad, CA, USA) with enzyme-linked immunosorbent assays according to the recommendations of the manufacturer. The absorbance was measured at 450 nm using a microliter ELISA reader (Versa Max Molecular Devices, Sunnyvale, CA, USA). Samples were then analyzed with Versa Max microplate reader using SoftMaxPro5 software (Versa Max Molecular Devices, Sunnyvale, CA, USA). ELISA results were given as a percentage of the total protein concentration of the samples.

### 2.8. Real-Time Polymerase Chain Reaction (PCR) Assay

At the end of the 12-week etanercept administration, aortic samples were collected to determine the levels of TNF-α and eNOS. The gene expression levels were detected by real-time PCR. Total RNA was isolated by a High Pure RNA Isolation Kit (Roche, Mannheim, Germany) and then RNA concentrations were measured with the Nanodrop Spectrophotometer (Nanodrop Technology, Cambridge). 1 µg of total RNA was reverse transcribed into cDNA by Transcriptor High Fidelity cDNA Synthesis Kit (Roche), according to the instruction manuals. Target gene amplifications were performed with gene-specific probes (Universal ProbeLibrary, Roche), according to the manufacturer’s recommendations on the instrument, LightCycler 480II (Roche, Diagnostic Rotkreuz, Switzerland). The primer sequences matched the indications of Universal Probe Library (Roche). The PCR conditions were as follows (two-step PCR): incubation for 15 s at 94 °C and 1 min at 60 °C for 45 cycles, annealing temperature at 60 °C. Primers used for real time PCR as follows: TNF-α forward 5′-TGACCCTCACCGATACAACA and reverse 3′-CGGGTGTCTAGATCCATGC; eNOS forward 5′-GAACTTCGGGGTGATCG and reverse 3′-GGGCTTGTCACTCGAGTTTT. Primers (TNF-α and eNOS, Iontek İstanbul, Turkey) and other supplements were added according to the manufacturer’s instructions. The target and reference genes were amplified in the same wells. The reaction mixture, lacking cDNA, was used as a negative control in each run. In addition, cDNA reaction mix without template was used as a negative control. The comparative amount of gene expression of eNOS—otherwise known as nitric oxide synthase 3 (NOS_3_) and TNF-α combined with the housekeeping gene Actin, beta (Actb), and GADPH, identified as endogenous control and a positive calibrator—offered a basis for stabilizing sample-to-sample disparities. The Light Cycler software (Roche, Mannheim, Germany) was utilized for the examination of real-time PCR results.

### 2.9. Statistical Analysis

Data were presented as mean ± standard deviation of the mean (SD). The contractile force was expressed in milligrams of developed tension. Relaxation was presented as a percentage of the pre-contractile response to phenylephrine. Maximum response (Emax) and pD2 (−log EC50) was calculated using software. The cumulative concentration–response curve data were fit to a four-parameter logistic equation: E = Emax/1 + (EC50/[D]n), where E is the observed effect in grams of tension, Emax is the calculated maximal effect, [D] is the concentration of agonist, EC50 is the [D] at 0.5 Emax, and *n* is the slope factor parameter. Differences between experimental groups were assessed using one-way ANOVA followed by Bonferroni’s post hoc test. For immunohistochemical analysis the Kolmogorov–Smirnov test was used to assess the normality of the data. Comparisons of multiple samples were performed using one-way ANOVA with a post-hoc Tukey test. *p* < 0.005 was assumed to be statistically significant.

## 3. Results

### 3.1. Etanercept Treatment Reduced Body Weight

**Figure 1:**
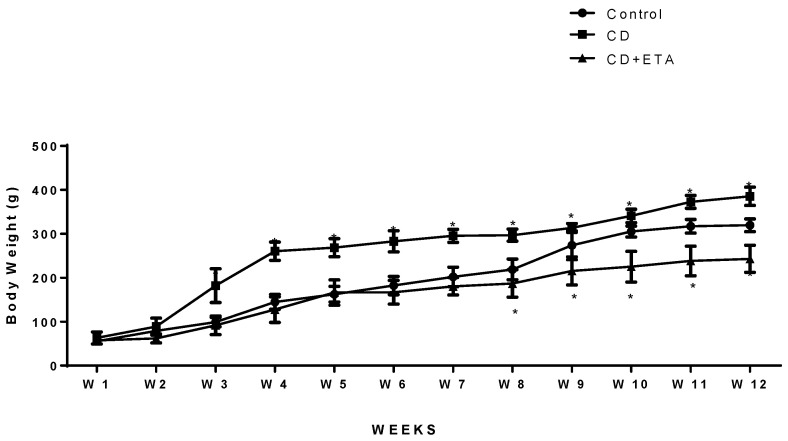
Bodyweight (g) of rats fed a standard pelleted diet (control), cafeteria diet (CD), or cafeteria diet with etanercept treatment (CD + ETA) during 12 weeks (*n* = 10). Data are expressed as the mean ± S.D. * Statistical difference compared to the control group. * *p* < 0.05 is considered significant.

No significant differences were revealed in the body weight between the groups at the onset of the experiments (*p* > 0.05; Figure 1, Table 1). At the end of the experimental period, the cafeteria diet induced a significant increase in body weight (CD group) compared with the standard diet (Figure 1, Table 1). In addition, the bodyweight gains of rats were statistically higher when compared with the standard diet-fed rats. Etanercept administration decreased final body weight compared to the untreated CD groups (*p* < 0.0001; Figure 1), although food intake was not affected (Table 1).

### 3.2. Etanercept Treatment Improved Vascular Contractility and Vasorelaxation

The study groups’ aortas showed comparable contractile responses to 80 mM KCl. As illustrated in Table 2, no significant variation was recorded between the maximum responses of the rings belonging to the control, CD and CD + ETA groups (*p* > 0.05).

**Figure 2:**
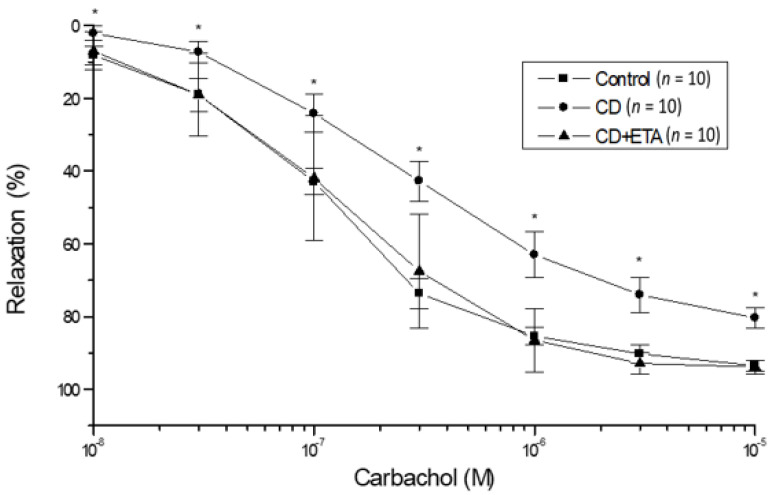
Carbachol concentration-response curves from isolated thoracic aortic rings precontracted with 3 × 10^−6^–10^−5^ M phenylephrine. Each data point is expressed as a percentage of the contraction induced by phenylephrine and is given as the mean ± S.D. The numbers in parentheses indicate the number of preparations used. * *p* < 0.05, statistically different from the response of aortic rings from control and CD + ETA groups. Control (not exposed to CD), CD (exposed to CD during 12 weeks), and CD + ETA (exposed to CD and treated with subcutaneous injections of etanercept (0.8 mg/kg/week) during 12 weeks). Control group was fed with standard pellet diet.

The endothelium-dependent vasorelaxation to carbachol was subject to examination in thoracic aortas from all experimental groups for the purpose of evaluating the endothelial function. Carbachol stimulated concentration-dependent relaxation, which was found to be considerably reduced in aortic tissues from the CD group in comparison with the control group. (*p* < 0.05, Figure 2). The endothelial dysfunction in the CD group was emphasized as well by lower Emax and pD2 values when comparing carbachol curves (*p* < 0.05, Figure 2, Table 2). The long-term administration of etanercept significantly reinstated the aorta impairment in endothelium-dependent relaxation to carbachol from CD-fed animals (*p* < 0.05, Figure 2, Table 2). Emax and pD2 values were similar in control and CD + ETA groups (Figure 2, Table 2).

**Figure 3:**
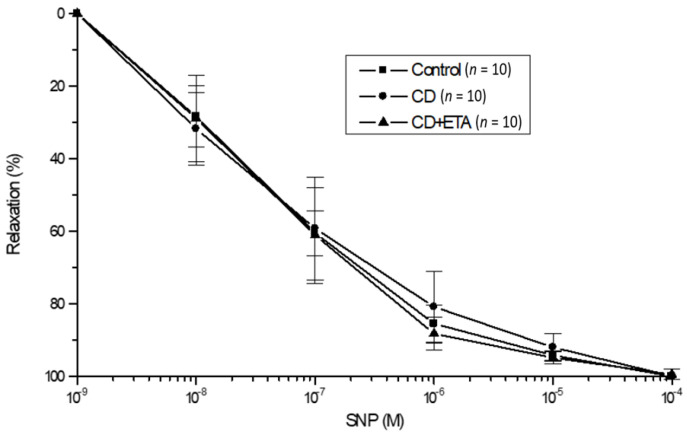
Sodium nitroprusside concentration-response curves in isolated thoracic aortic rings precontracted with 3 × 10^−6^–10^−5^ M phenylephrine. Each data point is expressed as a percentage of the contraction induced by phenylephrine and is given as the mean ± S.D. The numbers in parentheses indicate the number of preparations used. Control (not exposed to CD), CD (exposed to CD during 12 weeks), and CD + ETA (exposed to CD and treated with subcutaneous injections of etanercept (0.8 mg/kg/week) during 12 weeks). Control group was fed with standard pellet diet.

The experimental groups displayed no significant differences regarding endothelium-independent relaxation in response to NO donor SNP (10^−8^–10^−4^ M) in aortic rings. Moreover, no variations were found in Emax and pD2 values. (Figure 3, Table 2). Also, no substantial modifications were detected in the relaxation response to papaverine of any aortas from the groups.

### 3.3. Etanercept Treatment Corrected Alterations of Endothelial Nitric Oxide Synthase (eNOS) Immunoreactivity

**Figure 4:**
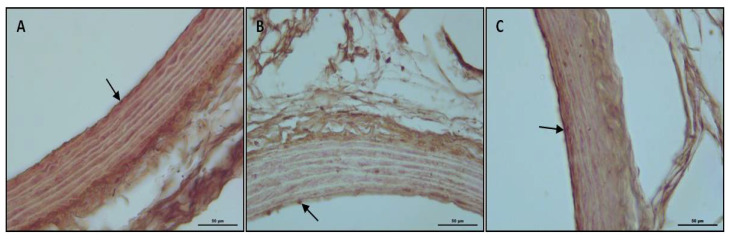
Representative light microscopy of the control, CD, and CD + ETA groups in the endothelium of the thoracic aorta (*n* = 10). Decreased endothelial nitric oxide synthase (eNOS) immunoreactivity in the CD group (**B**) compared with the control group (**A**), and increased eNOS immunoreactivity in the CD + ETA group (**C**) compared with the CD group (**B**). Control (not exposed to CD), CD (exposed to CD during 12 weeks), and CD + ETA (exposed to CD and treated with subcutaneous injections of etanercept (0.8 mg/kg/week) during 12 weeks). Control group was fed with standard pellet diet.

**Figure 5:**
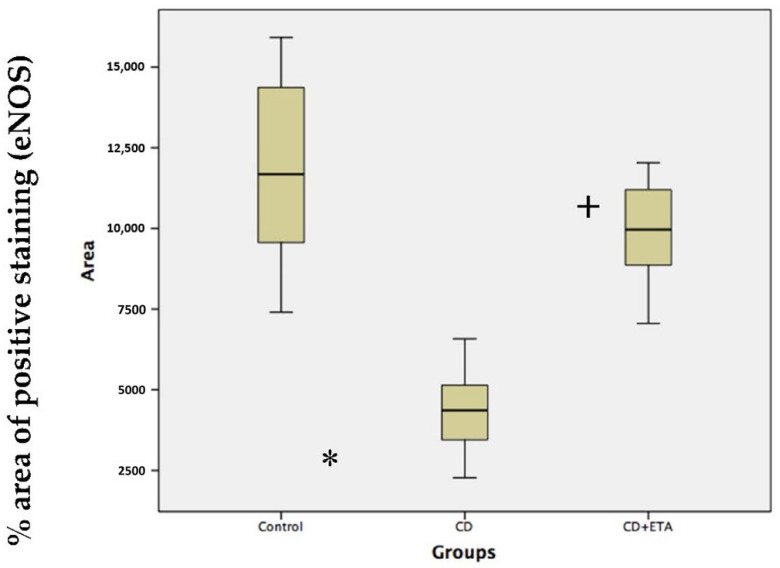
Analysis of immunohistochemistry staining using ImageJ. The immunopositivity was decreased in CD group compared to the control group. In the CD + ETA group, eNOS immunoreactivity was similar to that of the control group. * indicates significance from the control group at *p* < 0.05 comparability test, + indicates significance from the CD group at *p* < 0.05 probability test. Control (not exposed to CD), CD (exposed to CD during 12 weeks), and CD + ETA (exposed to CD and treated with subcutaneous injections of etanercept (0.8 mg/kg/week) during 12 weeks). Control group was fed with standard pellet diet.

eNOS immunoreactivity was found in arterial preparations from control rats (Figure 4, Table 3). Aortic tissue from CD group showed decreased immunopositivity of eNOS compared to the control group (*p* < 0.05, Figure 4, Table 3). Etanercept treatment induced a marked increment of aortic eNOS immunoreactivity in the vascular wall compared with the CD group (*p* < 0.05, Figure 4, Table 3). The analysis of immunohistochemistry staining to describe immunoreactivity was performed by using ImageJ (Figure 5).

### 3.4. Etanercept Improved Alterations of Serum and Aortic Inflammatory Markers and Aortic eNOS Expressions

**Figure 6:**
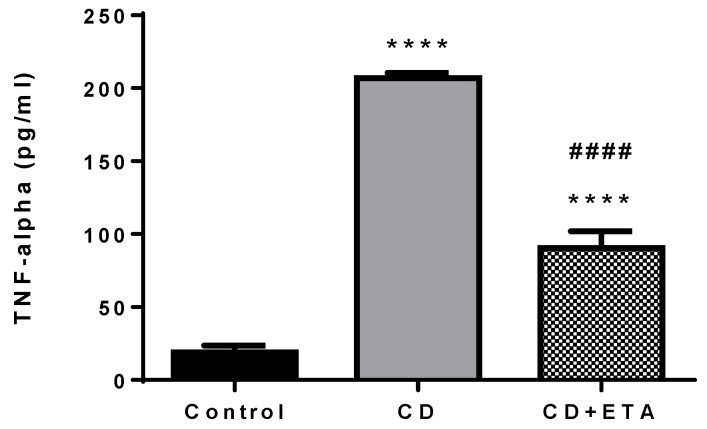
Serum TNF-α levels in control and CD-fed rats treated with or without etanercept (*n* = 10). **** *p* < 0.0001, statistically different from the control group and #### *p* < 0.0001, statistically different from the CD group. Control (not exposed to CD), CD (exposed to CD during 12 weeks), and CD + ETA (exposed to CD and treated with subcutaneous injections of etanercept (0.8 mg/kg/week) during 12 weeks). Control group was fed with standard pellet diet.

Serum concentration of TNF-α was significantly higher in CD group than in control group (*p* < 0.0001), and etanercept treatment significantly suppressed TNF-α levels in CD + ETA group compared to the CD group (*p* < 0.0001, Figure 6).

**Figure 7:**
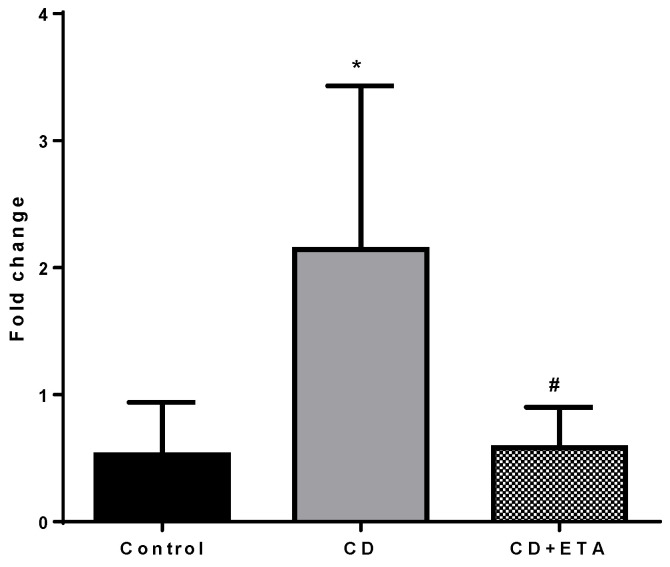
Aortic TNF-α expressions in control and CD-fed rats treated with or without etanercept (*n* = 10). * *p* < 0.0001, statistically different from the control group and # *p* < 0.0001, statistically different from the CD group. Control (not exposed to CD), CD (exposed to CD during 12 weeks), and CD + ETA (exposed to CD and treated with subcutaneous injections of etanercept (0.8 mg/kg/week) during 12 weeks). Control group was fed with standard pellet diet.

**Figure 8:**
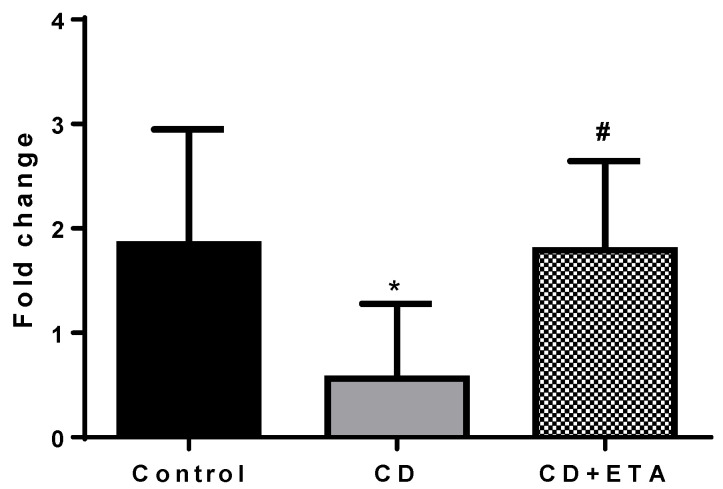
Aortic eNOS expressions in control and CD-fed rats treated with or without etanercept (*n* = 10). * *p* < 0.0001, statistically different from the control group and # *p* < 0.0001, statistically different from the CD group. Control (not exposed to CD), CD (exposed to CD during 12 weeks), and CD + ETA (exposed to CD and treated with subcutaneous injections of etanercept (0.8 mg/kg/week) during 12 weeks). Control group was fed with standard pellet diet.

The gene expression of TNF-α and eNOS in aortic tissue was higher in CD group (*p* < 0.0001, Figure 7 and Figure 8, respectively) and no significant difference in CD + ETA group compared to the control group (Figure 7 and Figure 8).

## 4. Discussion

This is the first report showing that chronic etanercept administration prevented endothelium-dependent vascular dysfunction and body weight gain in CD-fed rats. Although the role of TNF-α on vascular impairments has been reported, the novel findings of the current study revealed that anti-TNF-α treatment abrogated obesity-induced vascular dysfunction in rats by suppressing inflammation, indicating that anti-inflammatory agents might be considered as a candidate for treatment of cardiovascular disorders associated with obesity. In this study, our results showed that NO-mediated vasorelaxant responses to carbachol were impaired in CD-fed rats compared with control rats. This impairment was associated with increased concentrations of TNF-α. Moreover, aortic eNOS expression significantly decreased in rats exposed to CD compared with the control group. However, 12-week chronic etanercept treatment significantly restored endothelium-dependent vasorelaxation and resulted in decreased serum levels of TNF-α and increased eNOS expression in rats exposed to CD. SNP-, an NO donor, induced endothelium-independent vasorelaxation was not affected. 

CD is a diet-induced obesity model used in experimental studies to mimic the food intake of people in modern Western societies. In this model, body weight gain is observed not only after chronic diet treatment [21] but also in the early stages of obesity onset [22]. Consistent with this observation, our data demonstrated that early body weight gain in rats was reached after 3 weeks of CD treatment and a 12-week CD significantly enhanced body weight in Wistar rats, indicating that a model of obesity was established successfully.

Many of the clinical and experimental studies demonstrated that endothelial function was decreased by obesity which is an important risk factor for cardiovascular disease [3,17]. These data are in accordance with this study in which CD-induced obesity caused an impairment of carbachol-induced endothelium-dependent vasodilatation. It is well established that in intact endothelium, carbachol or acetylcholine causes an endothelium-dependent vasorelaxation in vascular smooth muscle cells that are precontracted with higher concentrations of noradrenaline, potassium, or other vasoconstrictor agents such as phenylephrine [23]. In this context, the impairment of endothelium-dependent vasorelaxation of vascular smooth muscle in this study could be associated with one of the following mechanisms: the reduction of NO sensitivity, the impairment of the relaxation of vascular smooth muscle, or the alteration of the NO/cyclic guanosine monophosphate (cGMP) pathway. However, the maximum vasorelaxation induced by SNP and papaverine in endothelium-intact aortic tissues did not change significantly between rings obtained from rats fed a standard diet and those from animals exposed to CD. Since SNP acts to release NO directly within vascular smooth muscle cells after its metabolism, these mechanisms appear not to be involved in the impairment of endothelium-dependent vasorelaxation induced by obesity. Once released by SNP, NO binds to the normally reduced heme iron group on soluble guanylate cyclase (sGC) and enhances the generation of cGMP, which causes vasodilation [24]. In this regard, these findings indicate that diet-induced obesity did not alter the smooth muscle response to NO. Additionally, cGMP-dependent mechanisms do not appear to contribute to the development of obesity-induced dysfunction in vascular tissue. Moreover, the contractile responses to 80 mM KCl were similar among all groups, demonstrating that the contractile mechanisms of vascular smooth muscle did not alter by diet-induced obesity. Our study has also shown that the pre-contractile tone induced by phenylephrine was similar in thoracic aortic rings derived from rats fed a standard diet and CD, confirming that the relaxation differences were not associated with the degree of precontraction. Considering these findings data suggests that the impairment of endothelium-dependent vasorelaxation induced by CD could be due to the reduction of NO bioavailability by decreasing either the production or release of NO in the vascular tissue. Consistently, our data showed that vascular eNOS expression was decreased in CD-fed rats, indicating reduced NO production. Also, previous studies in different kind of experimental obesity models reported that obesity caused impairment of the carbachol or acetylcholine-induced endothelium-dependent relaxation with no change in the endothelium-independent vasorelaxation in response to SNP in aorta, demonstrating the decreased NO bioavailability [25,26]. Furthermore, it has been reported that vascular basal NO production decreased in obese patients [27]. All these data including ours indicate that obesity is strongly associated with endothelial dysfunction with the loss of NO bioavailability.

There is a growing body of evidence suggesting that TNF-α-mediated inflammation plays a crucial role in the development of endothelial dysfunction. Several studies demonstrated that TNF-α, is one of the proinflammatory cytokines, impaired NO-dependent vasorelaxations in the endothelium of various vascular tissues [28,29,30] and decreased NO bioavailability by inducing the formation of ROS [31] or diminishing the production of NO [32]. Moreover, expression of eNOS, which plays the main role in endothelial NO production, was decreased by the direct effects of TNF-α in vascular tissue [33] and TNF-α reduced eNOS expression in endothelial cells by inhibiting gene promoter activity [34]. In addition, it has been reported that overproduction of TNF-α and the other endogenous proinflammatory cytokines play a substantial role in the pathophysiology of endothelial dysfunction observed in obesity, suggesting that obesity is a low-grade inflammatory disease. Accordingly, in this study, we observed elevated serum concentrations of TNF-α in CD-fed rats, indicating that TNF-α might mediate obesity-induced endothelial dysfunction. Our data were also compatible with previous publications, which reported increased circulating levels of TNF-α associated with obesity not only in rodents but also in obese patients [35,36]. Furthermore, a marked upregulation of TNF-α protein expression in vascular tissue was documented in obese animals and humans that have impaired endothelium-dependent vasorelaxations [18,35]. One group demonstrated that Ach-induced NO production and eNOS expression in isolated coronary arteries from obese Zucker rats markedly decreased compared with the lean control rats, via the elevated levels of circulating and protein expression of TNF-α [37]. In line with these data, we showed that TNF-α blockade with chronic etanercept treatment prevented obesity-induced endothelial dysfunction by increasing thoracic aortic eNOS expression and decreased serum concentrations of TNF-α in CD-fed rat, indicating the role of TNF-α in endothelial dysfunction related with obesity, and the close relationship between obesity and inflammation.

Based on the crucial role of TNF-α in improving endothelial dysfunction, the effects of different agents that block the action of TNF-α have been evaluated in different cardiovascular disorders and inflammatory conditions. Chronic treatment with infliximab, an anti-TNF-α antibody, prevented endothelial dysfunction of the brachial artery in patients with rheumatoid arthritis (RA) [38], systemic vasculitis [39] and Crohn’s disease [40]. Anti-TNF-α therapy with etanercept was also able to improve endothelial function in patients with RA [41] and advanced heart failure [42]. We previously reported that both etanercept and infliximab treatment ameliorated endothelial dysfunction related with stress [43,44]. Moreover, similar with our findings, infliximab in vitro improved endothelium-dependent vasorelaxation in small arteries of obese patients [18] and its infusion also ameliorated NO-dependent vasodilatation in the brachial artery in obese patients [45]. However, in contrast with our results, Dominguez et al. [46] reported that no improvement in endothelium-dependent vasodilation was observed in obese men treated with etanercept. However, in that study, patients received 4 weeks of etanercept treatment, which could be not sufficient to reverse endothelial dysfunction in obese patients and more importantly, people who initiated etanercept treatment were ‘already obese’ unlike our study. In this study, we fed Wistar rats with CD for 12 weeks to establish the obesity model and treated animals with etanercept during the CD treatment, not after obesity had been developed. Therefore, to our knowledge this is the first reported study showing evidence that TNF-α blockade with etanercept protects against endothelial dysfunction related with diet-induced obesity.

Finally, feeding the CD to rats resulted in significant increases in body weight compared with rats fed a standard diet. Although we did not characterize the body composition of animals tested, it can be assumed that most of the weight gain was because of increased adipose tissue. Increased body weight and adipose tissue may also lead to insulin resistance, and thus the lack of measurements of insulin and/or glucose levels of the animals may be considered a major limitation of our study. However, it can be suggested that our results might be secondary to obesity or obesity-induced insulin resistance. Also, surprisingly we found that anti-TNF-α treatment with etanercept induced significant weight loss in tested animals compared to CD-fed animals. This effect could be due to the blockade of TNF-α, which exerts its activity by two receptors: TNF-α receptor 1 (TNFR1) and TNF-α receptor 2 (TNFR2) [47]. Several studies revealed that both blockade and deletion of TNFR1 protected against diet-induced obesity [48,49]. Moreover, diet-induced obesity was ameliorated in mice lacking TNFR2 [50]. These findings suggested that TNF-α plays a crucial role in the development of obesity. In the current study, etanercept treatment prevented body weight gain in CD-fed rats, supporting the idea that anti-TNF-α compounds could be useful as therapeutic agents to prevent diet-induced obesity. 

The metabolic imbalances related to diet are emerging as major drivers of obesity-related cancer including alterations in growth factor signaling, inflammation and angiogenesis. A strong link between obesity and cancer risk and/or poor prognosis has been established in the epidemiological and preclinical literature [51]. Mechanisms of tumorigenesis, including inflammation and angiogenesis, may be influenced by specific dietary elements [52].

CAF diet induces hyperphagia and metabolic syndrome (MS) better than other diets. Indeed, endothelium-dependent vasorelaxation is strictly linked to non-alcoholic fatty liver disease (NAFLD) and is mainly due to insulin resistance. On those bases there are no data on hepatic steatosis of animals treated with a CAF diet. One other main limitation to study are the common mechanisms underlying the two pathologies (endothelial and vascular reactivity and NAFLD), and in both insulin resistance plays a central role.

Dealing with insulin resistance and oxido-reductive imbalance, to give those outside this field of knowledge a broader view of the issue, sirtuin 4 can play a central role as accelerating Ang II-induced pathological cardiac hypertrophy by inhibiting manganese superoxide dismutase activity and preventing hypoxia-induced apoptosis in H9c2 cardiomyoblast cells. Circulating levels of sirtuin 4, a potential marker of oxidative metabolism, is related to coronary artery disease in obese patients suffering from NAFLD, with normal or slightly increased liver enzymes.

## 5. Conclusions

Our study demonstrated that etanercept treatment prevented endothelium-dependent vascular dysfunction and body weight gain caused by CD. Thus, TNF-α-mediated inflammation may have an important role in endothelial dysfunction and body weight increment related to CD consumption.

In conclusion, the present data suggest that chronic etanercept treatment is likely able to prevent body weight gain and the development of endothelial dysfunction by restoring eNOS expression and reducing serum levels of TNF-α in CD-fed rats, indicating that TNF-α-mediated inflammation has a crucial role on endothelial dysfunction and body weight increment related with CD consumption.

The beneficial effects of etanercept and its specific mechanisms require further evaluation in future studies.

## 6. Patents

There are no patents resulting from the work reported in this manuscript.

## Figures and Tables

**Table 1:** Body-related and food intake measurements of the three dietary experimental groups.

	Control (*n* = 10)	CD (*n* = 10)	CD + ETA (*n* = 10)
**Initial body weight (g)**	55.1 ± 4.65	61.10 ± 9.23	58.99 ± 10.46
**Final body weight (g)**	265.5 ± 10.11	334.9 ± 7.87 *	293.9 ± 18.68 *
**Body weight gain at 12 weeks (g)**	210.4 ± 7.55	273.5 ± 4.33 *	234.9 ± 10.65 *
**kcal intake/day (kcal)**	35.17 ± 1.33	131.7 ± 9.01 *	134.1 ± 11.34 *

Results are expressed as mean ± S.D. * Statistical difference compared to the control group. * *p* < 0.05 is considered significant. Control (not exposed to CD), CD (exposed to CD during 12 weeks), and CD + ETA (exposed to CD and treated with subcutaneous injections of etanercept (0.8 mg/kg/week) during 12 weeks). Control group was fed with standard pellet diet.

**Table 2:** Emax (% of 3 × 10^−6^–10^−5^ M phenylephrine) values for carbachol, sodium nitroprusside; Emax values (mg) for 80 mM KCl; Emax values for 10^−5^–10^−4^ M papaverine and pD2 values (-log EC50) for carbachol and sodium nitroprusside (SNP) in rings of thoracic aorta were obtained from all three groups of rats.

	Control (*n* = 10)	CD (*n* = 10)	CD + ETA (*n* = 10)
**Carbachol** *E*_max_ pD_2_	93.50 ± 1.67 6.95 ± 0.06	80.38 ± 3.25 * 6.66 ± 0.13 *	93.60 ± 1.90 6.92 ± 0.28
**SNP** *E*_max_ pD_2_	100.00 ± 0.00 7.34 ± 0.25	100.00 ± 0.00 7.26 ± 0.51	99.50 ± 1.58 7.36 ± 0.41
**KCl** *E*_max_	904.38 ± 250.87	999.80 ± 260.92	926.75 ± 243.16
**Papaverine** *E*_max_	100 ± 0.00	100 ± 0.00	100 ± 0.00

Values are arithmetic means ± S.D, *n* = the number of thoracic aortic rings used. Each ring was obtained from different rats. * *p* < 0.0001, statistically different from control rats. * *p* < 0.05, statistically different from the response of aortic rings from control rats. Control (not exposed to CD), CD (exposed to CD during 12 weeks), and CD + ETA (exposed to CD and treated with subcutaneous injections of etanercept (0.8 mg/kg/week) during 12 weeks). Control group was fed with standard pellet diet.

**Table 3:** Semi-quantitative distribution of eNOS—immunoreactivity of aorta in rats.

Animal	Control	CD	CD + ETA
1 2	2+ 3+ 2+ 2+ 3+ 2+ 2+ 2+ 2+ 1+	1+ 2+ 1+ 1+ 2+ 1+ 1+ 1+ 2+ 1+	2+ 2+ 3+ 2+ 2+ 1+ 2+ 2+ 3+ 3+
3 4 5 6 7 8 9 10			

The staining intensity was classified no expression (-), very (1+), moderate (2+), strong (3+) to very strong (4+) expression. The immunopositivity was decreased in CD group compared to the control group (*p* < 0.05, Kruskal-Wallis Test.) In the CD+ETA group, eNOS immunoreactivity was similar to that of the control group.

## Data Availability

Data available on request due to restrictions regarding privacy. The data presented in this study are available on request from the corresponding author. The data are not publicly available due to privacy.

## References

[1] Calabrò P., Golia E., Maddaloni V., Malvezzi M., Casillo B., Marotta C., Calabrò R., Golino P. (2008). Adipose tissue-mediated inflammation: The missing link between obesity and cardiovascular disease?. Intern. Emerg. Med..

[2] Al Suwaidi J., Higano S.T., Holmes D.R., Lennon R., Lerman A. (2001). Obesity is independently associated with coronary endothelial dysfunction in patients with normal or mildly diseased coronary arteries. J. Am. Coll. Cardiol..

[3] Perticone F., Ceravolo R., Candigliota M., Ventura G., Iacopino S., Sinopoli F., Mattioli P.L. (2001). Obesity and body fat distribution induce endothelial dysfunction by oxidative stress: Protective effect of vitamin C. Diabetes.

[4] Grassi G., Seravalle G., Scopelliti F., Dell’Oro R., Fattori L., Quarti-Trevano F., Brambilla G., Schiffrin E., Mancia G. (2010). Structural and Functional Alterations of Subcutaneous Small Resistance Arteries in Severe Human Obesity. Obesity.

[5] Han L., Yu Y., Sun X., Wang B. (2012). Exendin-4 directly improves endothelial dysfunction in isolated aortas from obese rats through the cAMP or AMPK-eNOS pathways. Diabetes Res. Clin. Pract..

[6] Behrendt D., Ganz P. (2002). Endothelial function: From vascular biology to clinical applications. Am. J. Cardiol..

[7] Williams I., Wheatcroft S., Shah A., Kearney M. (2002). Obesity, atherosclerosis and the vascular endothelium: Mechanisms of reduced nitric oxide bioavailability in obese humans. Int. J. Obes..

[8] Zhang H., Park Y., Wu J., Chen X.P., Lee S., Yang J., Dellsperger K.C., Zhang C. (2009). Role of TNF-α in vascular dysfunction. Clin. Sci..

[9] Berk B.C., Abe J.-I., Min W., Surapisitchat J., Yan C. (2008). Endothelial Atheroprotective and Anti-inflammatory Mechanisms. Ann. N. Y. Acad. Sci..

[10] Ferrari R. (1998). Tumor necrosis factor in CHF: A double facet cytokine. Cardiovasc. Res..

[11] Wang M., Tsai B.M., Reiger K.M., Brown J.W., Meldrum D.R. (1998). Human myocardial tissue TNFalpha expression following acute global ischemia in vivo. J. Mol. Cell Cardiol..

[12] Chia S., Qadan M., Newton R., Ludlam C.A., Fox K., Newby D.E. (2003). Intra-Arterial Tumor Necrosis Factor-α Impairs Endothelium-Dependent Vasodilatation and Stimulates Local Tissue Plasminogen Activator Release in Humans. Arter. Thromb. Vasc. Biol..

[13] Yoshizumi M., Perrella M.A., Burnett J.C., Lee M.E. (1993). Tumor necrosis factor down regulates an endothelial nitric oxide synthase mRNA by shortening its half-life. Circ. Res..

[14] Neumann P., Gertzberg N., Johnson A. (2004). TNF-α induces a decrease in eNOS promoter activity. Am. J. Physiol. Cell. Mol. Physiol..

[15] López I.P., Marti A., Milagro F.I., Zulet M.D.L.A., Moreno-Aliaga M.J., Martinez J.A., De Miguel C. (2003). DNA Microarray Analysis of Genes Differentially Expressed in Diet-Induced (Cafeteria) Obese Rats. Obes. Res..

[16] Dandona P., Weinstock R., Thusu K., Abdel-Rahman E., Aljada A., Wadden T. (1998). Tumor Necrosis Factor-α in Sera of Obese Patients: Fall with Weight Loss. J. Clin. Endocrinol. Metab..

[17] Justo M.L., Candiracci M., Dantas A.P., de Sotomayor M.A., Parrado J., Vila E., Herrera M.D., Rodriguez-Rodriguez R. (2013). Rice bran enzymatic extract restores endothelial function and vascular contractility in obese rats by reducing vascular inflammation and oxidative stress. J. Nutr. Biochem..

[18] Virdis A., Santini F., Colucci R., Duranti E., Salvetti G., Rugani I., Segnani C., Anselmino M., Bernardini N., Blandizzi C. (2011). Vascular Generation of Tumor Necrosis Factor-α Reduces Nitric Oxide Availability in Small Arteries from Visceral Fat of Obese Patients. J. Am. Coll. Cardiol..

[19] Utkan T., Yazir Y., Karson A., Bayramgurler D. (2015). Etanercept Improves Cognitive Performance and Increases eNOS and BDNF Expression During Experimental Vascular Dementia in Streptozotocin- induced Diabetes. Curr. Neurovascular Res..

[20] Garcia-Diaz D.F., Campion J., Milagro F.I., Paternain L., Solomon A., Martinez J.A. (2009). Ascorbic acid oral treatment modifies lipolytic response and behavioural activity but not glucocorticoid metabolism in cafeteria diet-fed rats. Acta Physiol..

[21] García-Díaz D., Campión J., Milagro F.I., Martínez J.A. (2007). Adiposity dependent apelin gene expression: Relationships with oxidative and inflammation markers. Mol. Cell. Biochem..

[22] López I., Milagro F., Martí A., Moreno-Aliaga M., Martínez J., De Miguel C. (2004). Gene expression changes in rat white adipose tissue after a high-fat diet determined by differential display. Biochem. Biophys. Res. Commun..

[23] Furchgott R.F. (1983). Role of endothelium in responses of vascular smooth muscle. Circ. Res..

[24] Moncada S., Palmer R.M., Higgs E.A. (1991). Nitric oxide: Physiology, pathophysiology, and pharmacology. Pharmacol. Rev..

[25] Kobayasi R., Akamine E.H., Davel A.P., Rodrigues M.A., Carvalho C.R., Rossoni L.V. (2010). Oxidative stress and inflammatory mediators contribute to endothelial dysfunction in high-fat diet-induced obesity in mice. J. Hypertens..

[26] López-Canales J.S., Cuenca J.L., López-Canales O.A., Aguilar-Carrasco J.C., Aranda-Zepeda L., López-Sánchez P., Castillo-Henkel E.F., López-Mayorga R.M., Valencia-Hernández I. (2015). Pharmacological characterization of mechanisms involved in the vasorelaxation produced by rosuvastatin in aortic rings from rats with a cafeteria-style diet. Clin. Exp. Pharmacol. Physiol..

[27] Virdis A., Duranti E., Rossi C., Dell’Agnello U., Santini E., Anselmino M., Chiarugi M., Taddei S., Solini A. (2015). Tumour necrosis factor-alpha participates on the endothelin-1/nitric oxide imbalance in small arteries from obese patients: Role of perivascular adipose tissue. Eur. Heart J..

[28] Nyström T., Nygren A., Sjöholm Å. (2006). Increased levels of tumour necrosis factor-α (TNF-α) in patients with Type II diabetes mellitus after myocardial infarction are related to endothelial dysfunction. Clin. Sci..

[29] Gao X., Belmadani S., Picchi A., Xu X., Potter B.J., Tewari-Singh N., Capobianco S., Chilian W.M., Zhang C. (2007). Tumor Necrosis Factor-α Induces Endothelial Dysfunction in Lepr db Mice. Circulation.

[30] Zhang C., Xu X., Potter B.J., Wang W., Kuo L., Michael L., Bagby G.J., Chilian W.M. (2006). TNF-a contributes to endothelial dysfunction in ischemia/reperfusion injury. Arterioscler. Thromb. Vasc. Biol..

[31] Yang B., Rizzo V. (2007). TNF-α potentiates protein-tyrosine nitration through activation of NADPH oxidase and eNOS localized in membrane rafts and caveolae of bovine aortic endothelial cells. Am. J. Physiol. Circ. Physiol..

[32] Greenberg S., Xie J., Wang Y., Cai B., Kolls J., Nelson S., Hyman A., Summer W.R., Lippton H. (1993). Tumor necrosis factor-alpha inhibits endothelium-dependent relaxation. J. Appl. Physiol..

[33] Goodwin B.L., Pendleton L.C., Levy M.M., Solomonson L.P., Eichler D.C. (2007). Tumor necrosis factor-α reduces argininosuccinate synthase expression and nitric oxide production in aortic endothelial cells. Am. J. Physiol. Circ. Physiol..

[34] Anderson H., Rahmutula D., Gardner D.G. (2004). Tumor Necrosis Factor-α Inhibits Endothelial Nitric-oxide Synthase Gene Promoter Activity in Bovine Aortic Endothelial Cells. J. Biol. Chem..

[35] Nishimatsu H., Suzuki E., Takeda R., Takahashi M., Oba S., Kimura K., Nagano T., Hirata Y. (2008). Blockade of Endogenous Proinflammatory Cytokines Ameliorates Endothelial Dysfunction in Obese Zucker Rats. Hypertens. Res..

[36] Shin J., Kim S., Jeung M., Eun S., Woo C., Yoon S.-Y., Lee K.-H. (2008). Serum Adiponectin, C-Reactive Protein and TNF-α Levels in Obese Korean Children. J. Pediatr. Endocrinol. Metab..

[37] Picchi A., Gao X., Belmadani S., Potter B.J., Focardi M., Chilian W.M., Zhang C. (2006). Tumor Necrosis Factor-α Induces Endothelial Dysfunction in the Prediabetic Metabolic Syndrome. Circ. Res..

[38] Cardillo C., Schinzari F., Mores N., Mettimano M., Melina D., Zoli A., Ferraccioli G. (2006). Intravascular tumor necrosis factor? blockade reverses endothelial dysfunction in rheumatoid arthritis. Clin. Pharmacol. Ther..

[39] Booth A.D., Jayne D.R.W., Kharbanda R.K., McEniery C.M., Mackenzie I.S., Brown J., Wilkinson I.B. (2004). Infliximab improves endothelial dysfunction in systemic vasculitis: A model of vascular inflammation. Circulation.

[40] Schinzari F., Armuzzi A., De Pascalis B., Mores N., Tesauro M., Melina D., Cardillo C. (2008). Tumor necrosis factor-alpha antagonism improves endothelial dysfunction in patients with Crohn’s disease. Clin. Pharmacol. Ther..

[41] Bilsborough W., Keen H., Taylor A., O’driscoll G.J., Arnolda L., Green D.J. (2006). Anti-tumour necrosis factor-alpha therapy over conventional therapy improves endothelial function in adults with rheumatoid arthritis. Rheumatol. Int..

[42] Fichtlscherer S., Rössig L., Breuer S., Vasa M., Dimmeler S., Zeiher A.M. (2001). Tumor Necrosis Factor Antagonism with Etanercept Improves Systemic Endothelial Vasoreactivity in Patients with Advanced Heart Failure. Circulation.

[43] Bayramgurler D., Karson A., Yazir Y., Celikyurt I.K., Kurnaz S., Utkan T. (2013). The effect of etanercept on aortic nitric oxide-dependent vasorelaxation in an unpredictable chronic, mild stress model of depression in rats. Eur. J. Pharmacol..

[44] Demirtaş T., Utkan T., Karson A., Yazır Y., Bayramgürler D., Gacar N., Yazir Y. (2014). The Link Between Unpredictable Chronic Mild Stress Model for Depression and Vascular Inflammation?. Inflammation.

[45] Tesauro M., Schinzari F., Rovella V., Melina D., Mores N., Barini A., Mettimano M., Lauro D., Iantorno M., Quon M.J. (2008). Tumor Necrosis Factor- Antagonism Improves Vasodilation During Hyperinsulinemia in Metabolic Syndrome. Diabetes Care.

[46] Dominguez H., Storgaard H., Rask-Madsen C., Hermann T.S., Ihlemann N., Nielsen D.B., Spohr C., Kober L., Vaag A.A., Torp-Pedersen C. (2005). Metabolic and Vascular Effects of Tumor Necrosis Factor-α Blockade with Etanercept in Obese Patients with Type 2 Diabetes. J. Vasc. Res..

[47] Smith C.A., Farrah T., Goodwin R.G. (1994). The TNF receptor superfamily of cellular and viral proteins: Activation, costimulation, and death. Cell.

[48] Liang H., Yin B., Zhang H., Zhang S., Zeng Q., Wang J., Jiang X., Yuan L., Wang C.-Y., Li Z. (2008). Blockade of Tumor Necrosis Factor (TNF) Receptor Type 1-Mediated TNF-α Signaling Protected Wistar Rats from Diet-Induced Obesity and Insulin Resistance. Endocrinology.

[49] Romanatto T., Roman E.A., Arruda A.P., Denis R., Solon C., Milanski M., Moraes J.C., Bonfleur M.L., Degasperi G., Picardi P.K. (2009). Deletion of Tumor Necrosis Factor-α Receptor 1 (TNFR1) Protects against Diet-induced Obesity by Means of Increased Thermogenesis. J. Biol. Chem..

[50] Yamato M., Shiba T., Ide T., Seri N., Kudo W., Ando M., Yamada K.-I., Kinugawa S., Tsutsui H. (2011). High-fat diet–induced obesity and insulin resistance were ameliorated via enhanced fecal bile acid excretion in tumor necrosis factor-alpha receptor knockout mice. Mol. Cell. Biochem..

[51] Smith L.A., O’flanagan C.H., Bowers L.W., Allott E.H., Hursting S.D. (2018). Translating Mechanism-Based Strategies to Break the Obesity−Cancer Link: A Narrative Review. J. Acad. Nutr. Diet..

[52] Reynolds J.V., Donohoe C.L., Doyle S.L. (2010). Diet, obesity and cancer. Ir. J. Med Sci..

